# Supplementary material for: Large-scale cooperation driven by reputation, not fear of divine punishment
Source: R Soc Open Sci. 2019 Aug 21;6(8):190991. doi: 10.1098/rsos.190991 (PMC6731744; doi:10.1098/rsos.190991)
Supplement: Supplementary Material for"Large-scale cooperation driven by reputation,not fear of divine punishment" [file rsos190991supp1.docx]

**Electronic Supplementary Material for**

# ‘Large-scale cooperation driven by reputation, not fear of divine punishment’

Erhao Ge^$^ ^1^, Yuan Chen^$ 1^, Jiajia Wu^1^, Ruth Mace^*1,2^

$these authors contributed equally to this paper as co-first authors

*corresponding author: Ruth Mace. r.mace@ucl.ac.uk

^1^Lanzhou University, Life Sciences, 222 Tianshui South Rd, Lanzhou, Gansu Province,

730000, PRC

^2^Department of Anthropology, University College London, 14 Taviton Street,

London WC1H 0BW, UK

Supplementary Methods

1. Dice checking

Before conducting the dice game in the field, we did a dice quality test in the lab, after Gächter and Schulz ^1^. For a fair unbiased die, all numbers should be equally likely to occur. To test the quality of dice that were used in the experiment(n=5), we rolled each of them in a cup for 120 times and ran Chi-square tests(α=0.05) to see whether the empirical distribution is like the uniform distribution. From Supplementary Table S11, the distribution of each die is not significantly different from the uniform distribution, so we can conclude that the dice we used are unbiased.

2.Starting Endowment

According to the salary level and disposable personal income (DPI) in cities and villages in recent years from Gansu and Qinghai Province and National Bureau of Statistics ^2-5^, the average daily income for rural-dwellers is about 20 RMB, for town residents is about 70 RMB, we set the amount of money for participants to decide in each round as 10 RMB (locally called yuan), so each participant got in total 40 RMB to allocate in the experiment. In all, 508 people participated in the experiment, among which 4 gave up halfway in the game and 3 didn’t finish the questionnaire, so the number of our valid data is 501. In the end of the experiment, each participant got about 13￥on average.

3. Control Variable description.

**Offspring** (number of all living and dead children);

**Economic instability (**we measured economic instability based on the value of two questions:

*Do you worry that in the next month your household will have a time when it is not able to buy or produce enough food to eat?*

*Do you worry that in the next year your household will have a time when it is not able to buy or produce enough food to eat?*

The answer of the two questions are: 1) 0=No, 2) 1=Yes. The Economic instability index is the value of question about next month plus half the value of the question about next year and the reason is we reckon the urgency of next month is higher than that of next year.)

**Education** (0**=** illiteracy, 1= primary school, 2= junior high school, 3= senior high school,

4= Bachelor, 5= Master or above);

# Supplementary Figures

Supplementary Figure 1

**The regional map of the study area.** Study sites are in Gansu and Qinghai Provinces, Northwestern China. Site colour indicates dominant religion and shape indicates community size.


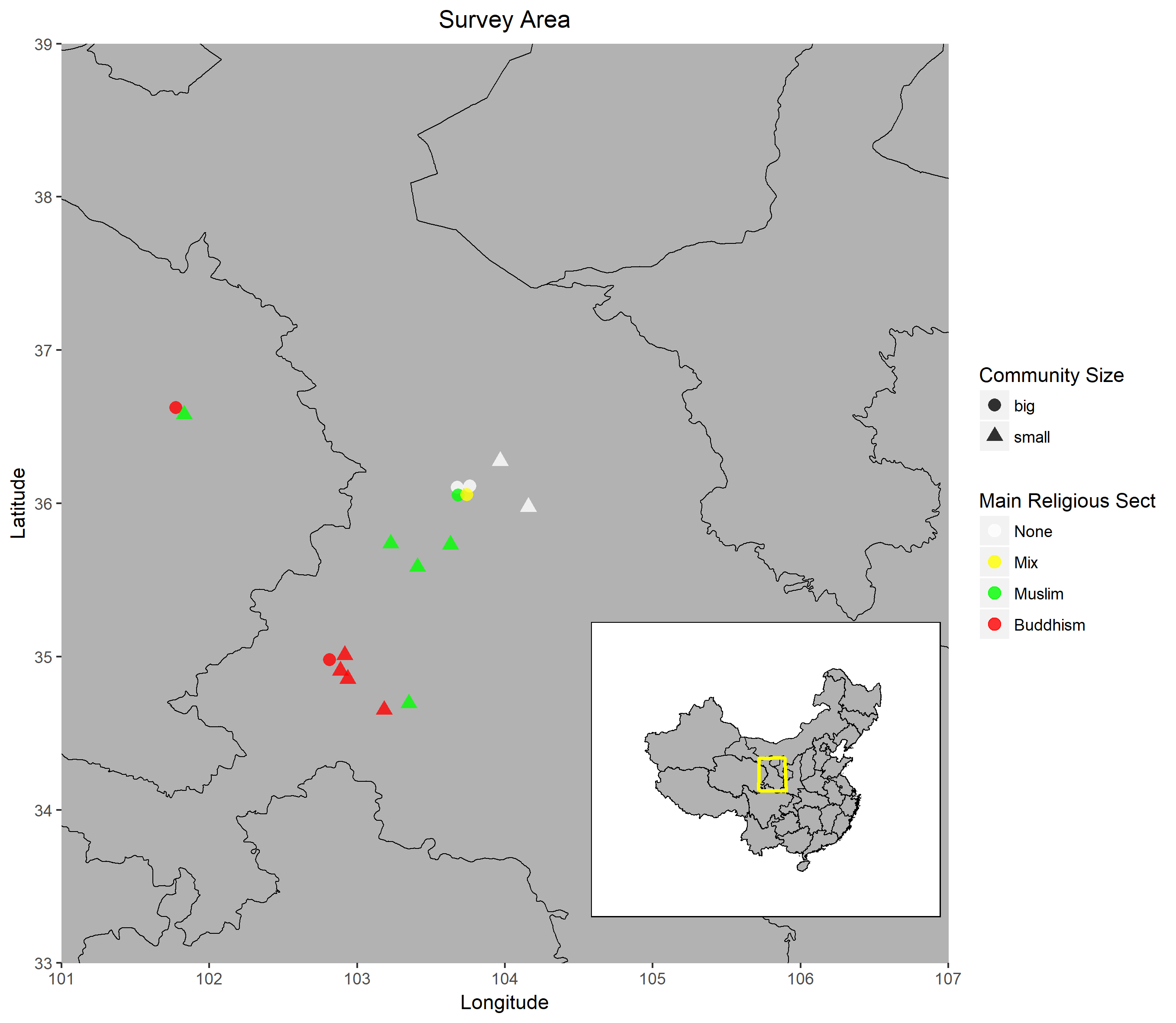


Supplementary Figure 2

**The cumulative dice earning distribution of seventeen communities**. Blue line represents the expected frequency of participant reporting of the higher rolls, which regarded as “Bending the rule”. Justified dishonesty is reporting the most advantageous of the two throws. (after Gächter and Schulz [1]).


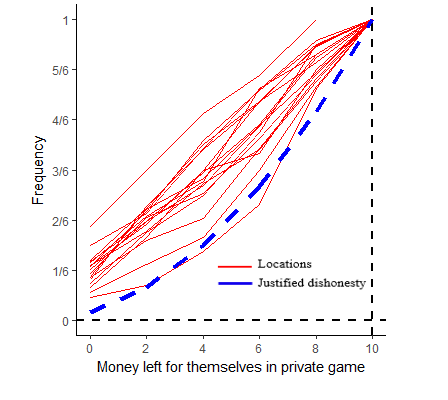


Supplementary Figure 3

**Proportion of choosing religious institution increases with higher economic instability in dice allocation game.** Each circle represents a survey location. The grey line represents the ordinary least-squares regression line (R²=0.819). The black line represents the regression line that controls average community education level and the proportion of female (R²=0.619, N=17).


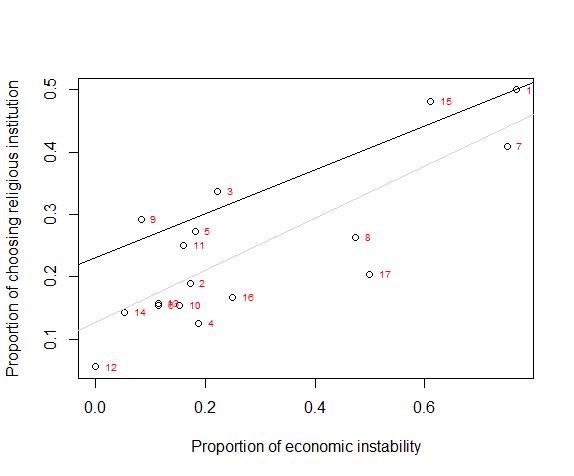


# Supplementary Tables

**Background Tables**

Supplementary Table 1

**Summary information for the communities studied.** Approximate community size is total number or approximate size range of households for the communities studied. The column Binary community division classifies the nature of each community in accordance with Approximate community size. N gives the number of participants drawn from each community.

| Site | N | Approximate community size | Binary community division | Main religion composition |
| --- | --- | --- | --- | --- |
| 1 | 28 | 130 | small | Muslim |
| 2 | 29 | 150 | small | Muslim |
| 3 | 101 | 150-300 | small | Buddhist |
| 4 | **8** | **More than 0.1 million** | **large** | **Mixed** |
| 5 | 11 | 4000 | small | Muslim |
| 6 | 13 | 260 | small | Muslim |
| 7 | 22 | 100 | small | Muslim |
| 8 | 19 | 1000 | small | Muslim |
| 9 | 12 | Less than 5000 | small | Buddhist |
| 10 | 13 | 400 | small | Buddhist |
| 11 | **28** | **More than 0.1 million** | **large** | **Buddhist** |
| 12 | 9 | 600 | small | atheist |
| 13 | 35 | Less than 10000 | small | atheist |
| 14 | **56** | **More than 0.1** **million** | **large** | **atheist** |
| 15 | **27** | **More than 0.1 million** | **large** | **Muslim** |
| 16 | **36** | **More than 0.1 million** | **large** | **Mixed** |
| 17 | **54** | **More than 0.1 million** | **large** | **Mixed** |

Supplementary Table 2

**S2 a)** The number of survey sites broken down by community size and main religious sect.

|  | Small community | large community | Sum |
| --- | --- | --- | --- |
| **Main Religious Sect** |  |  |  |
| None | 2 | 2 | 4 |
| Mixed | 0 | 1 | 1 |
| Buddhism | 4 | 2 | 6 |
| Muslim | 5 | 1 | 6 |
| Sum | 11 | 6 | 17 |

**S2 b)** Summary of participants we recruited from small and large communities with different religious beliefs (501 complete ones out of 504 participants).

|  | Small community | Large community | Sum |
| --- | --- | --- | --- |
| Atheist | 46 | 105 | 151 |
| Buddhist | 158 | 50 | 208 |
| Muslim | 88 | 54 | 142 |
| Sum | 292 | 209 | 501 |

Supplementary Table 3

Each participant could pick two institutions as recipients, and we found 43.5% participants chose Hope Project and 30.5% the Mother Water Cellar, while 14.5% chose the Buddhist monastery and 11.5% the Mosque. Participants with religion belief are significantly more likely to choose religious institution as their recipients.

**S3 a)** The distribution of specific choice by denomination.

|  | atheist | Muslim | Buddhist |
| --- | --- | --- | --- |
| Hope Projects | 139 | 114 | 184 |
| Buddhist monastery | 27 | 3 | 116 |
| Mosque | 10 | 104 | 0 |
| Mother Water Cellar | 126 | 63 | 116 |

**S3 b)** The distribution of religious, non-religious or mixed choices for each person by denomination

|  | atheist | Muslim | Buddhist |
| --- | --- | --- | --- |
| Both nonreligious choice | 117 | 37 | 92 |
| Mixed choice | 31 | 103 | 116 |
| Both religious choice | 3 | 2 | 0 |

**S3 c)** The distribution of nonreligious and religious choice by nonreligious and religious people.

|  | Atheist | Theist |
| --- | --- | --- |
| Nonreligious choice | 265 | 477 |
| Religious choice | 37 | 223 |

Supplementary Table 4

**Ecological difference between large and small communities**. Religious practice and divine punishment/reward are two components after PCA for the religiosity. Their scores are obtained by multiply loadings of a component with the standardised variables values and adding these products over variables.

|  | Small community  (n=292) | Large community  (n=209) |
| --- | --- | --- |
| Description | Mean (SD) | Mean (SD) |
| Sex ratio (male=1, female=2) | 1.34 | 1.45 |
| Mean age | 47.32(16.49) | 35.84(16.41) |
| Mean onlookers | 2.98(2.67) | 4.63(2.9) |
| Mean Education rank | 2.13(1.19) | 3.71(1.51) |
| Mean Dwelling time rank | 4.64(0.93) | 2.51(1.61) |
| Mean Economic instability | 0.29(0.56) | 0.29(0.56) |
| Choice ratio  (1=nonreligious,2=religious) | 1.28 | 1.22 |
| Mean Religious practice score | 0.17(1.05) | -0.23(0.86) |
| Mean Divine punishment/reward score | 0.15(0.92) | -0.22(1.06) |

Note that education and dwelling time are categorical variables: Dwelling time (1= 1-3years, 2= 3-5 years, 3= 5-10years, 4= 10-20 years, 5= more than 20years); **Education** (0**=** illiteracy, 1= primary school, 2= junior high school, 3= senior high school, 4= Bachelor, 5= Master or above)

**Religiosity Tables**

Supplementary Table 5

**Religious-related questions list** ^6^**.** The different variables have different ranges. All unknown and rejected responses are treated as missing values.

| **QUESTIONS** | **VARIABLE**  **SCALE** | **VARIABLE**  **NAME** |
| --- | --- | --- |
| 1.How important religion is to you in your life? | Unimportant (0)  A bit important (1)  Comparatively important (2)  Very important (3) | **Importance of religion** |
| 2.Have you donated any money or goods to religious organizations and/or religious individuals in the past twelve months? | No (0)  Yes (1) | **Religious**  **donation** |
| 3.How many religious activities have you done in the past twelve months? (give a list) | CONTINUES | **Religious Activities** |
| 4.How many the following situations will you seek help from religious institution?  1)You need help for advice on a personal matter  2) Someone in your family was sick  3) Get into financial difficulty | CONTINUES | **Appeal to religious institution** |
| 5.Do you believe that god/ the gods may punish people for doing a bad thing? | Don’t believe (0)  A bit of belief (1)  [Absolutely](file:///C:\Users\Geeh\AppData\Local\youdao\dict\Application\7.5.0.0\resultui\dict\?keyword=absolutely)[believe](file:///C:\Users\Geeh\AppData\Local\youdao\dict\Application\7.5.0.0\resultui\dict\?keyword=believe) (2) | **Belief in**  **God punishment** |
| 6.Do you believe that god/ the gods may reward people for doing a good thing? | Don’t believe (0)  A bit of belief (1)  [Absolutely](file:///C:\Users\Geeh\AppData\Local\youdao\dict\Application\7.5.0.0\resultui\dict\?keyword=absolutely)[believe](file:///C:\Users\Geeh\AppData\Local\youdao\dict\Application\7.5.0.0\resultui\dict\?keyword=believe) (2) | **Belief in**  **God**  **award** |
| 7.How many supernatural/ invisible things might exist in your personal view? (we will give a list) | CONTINUES | **Belief in invisible things** |
| 8.Do you believe that some people can harm other people through supernatural means? | Don’t believe (0)  A bit of belief (1)  [Absolutely](file:///C:\Users\Geeh\AppData\Local\youdao\dict\Application\7.5.0.0\resultui\dict\?keyword=absolutely)[believe](file:///C:\Users\Geeh\AppData\Local\youdao\dict\Application\7.5.0.0\resultui\dict\?keyword=believe) (2) | **Belief in supernatural power** |
| 9. Distance between survey spot and monastery/mosque (unit: km). | CONTINUES | **Religious institution distance** |

**Free Donation Game Tables**

Supplementary Table 6

**Parameter estimates in control and optimal fitting model of determinants of donating all the money to institutions in free donation game**. Survey sites as random effect. Significant effects are in bold.

|  | Free donation game(n=1002) | | | |  |
| --- | --- | --- | --- | --- | --- |
|  | Control model | | Control +CS+C+R | | |
|  | Est | Se | Est | Se | |
| Intercept | -0.506 | 0.495 | -0.732 | 0.489 | |
| Onlookers | -0.043 | 0.036 | -0.04 | 0.036 | |
| Gender(ref:man) | 0.181 | 0.169 | 0.252 | 0.171 | |
| Age | 0.003 | 0.006 | 0.004 | 0.006 | |
| Offspring | 0.04 | 0.084 | 0.021 | 0.085 | |
| Education | **0.380** | **0.076** | **0.422** | **0.078** | |
| Economic instability | **-0.430** | **0.141** | **-0.426** | **0.141** | |
| Community size(ref:small) |  |  | **-0.993** | **0.462** | |
| Choice (ref:nonreligious) |  |  | -0.111 | 0.182 | |
| Religious practice |  |  | **0.340** | **0.117** | |
| Divine punishment/reward |  |  | 0.082 | 0.079 | |
| Other supernatural belief |  |  | 0.136 | 0.081 | |
| *Random effect* |  |  |  |  | |
| Survey site | 0.991 | 0.996 | 0.639 | 0.799 | |

Supplementary Table 7

**A binomial model predicting whether donating all the endowment to institution and a count component (here, with a poisson distribution) predicting the magnitude of money donated.**

| **Free donation game** | | |
| --- | --- | --- |
|  | | |
|  | Dependent variable | |
|  |  | |
|  | Binary donation | Public donation amount |
|  | Zero | Count |
|  | | |
| Gender(ref:man) | 0.252 (0.171) | 0.060 (0.055) |
| Age | 0.004 (0.006) | 0.003 (0.002) |
| Offspring | 0.021 (0.085) | -0.047 (0.026) |
| Onlookers | -0.040 (0.036) | -0.001 (0.01) |
| Education | 0.422*** (0.078) | 0.083*** (0.023) |
| Economic instability | -0.426*** (0.141) | -0.056 (0.041) |
| Community size (ref:small) | -0.993** (0.462) | -0.195** (0.06) |
| Choice(ref:nonreligious) | -0.111 (0.182) | -0.032 (0.057) |
| Religious practice | 0.340*** (0.117) | 0.073** (0.028) |
| Divine punishment/reward | 0.082 (0.079) | -0.009 (0.024) |
| Other supernatural belief | 0.136* (0.081) | -0.029 (0.025) |
| Constant | -0.236 (0.497) | 1.494*** (0.141) |
|  | | |
| Observations | 1,002 | 353 |
|  | | |
| Note: | *p<0.1; **p<0.05; ***p<0.01 | |

Supplementary Table 8

**In which religious category is belief that divine punishment more punitive?**

**S8 a)** The multiple comparisons (Wilcoxon Rank Sum Test) of the extent to which participants in different religious denominations believe in God will punish you for bad acts. Group 2 is the group with the stronger belief in divine punishment in each comparison.

| group1(ref) | group2 | W | P |
| --- | --- | --- | --- |
| Atheist | Muslim | 6564.5 | <0.001 |
| Atheist | Buddhist | 11543.5 | <0.001 |
| Buddhist | Muslim | 13038 | <0.05 |

**S8 b)** The multiple comparisons of the extent to which participants in different religious denominations believe in God will reward you for good acts. Group 2 is the group with the stronger belief in divine reward.

| group1(ref) | group2 | W | p |
| --- | --- | --- | --- |
| Atheist | Muslim | 6954.5 | <0.001 |
| Atheist | Buddhist | 10281.5 | <0.001 |
| Buddhist | Muslim | 14536 | 0.7 |

Supplementary Table 9

**Whether participants who believe in more punitive religion are more likely to donating all the money to the chose institution.**

**S9 a)** Comparison of Buddhists and Muslims on the donation decision of the free donation game. Generalized linear multilevel model (binomial) was used with survey location as random effect.

|  | Estimate | SE | Z value | Pr(>\|z\|) |
| --- | --- | --- | --- | --- |
| (Intercept) | -0.64491 | 0.40585 | -1.589 | 0.11205 |
| Buddhist (ref:Muslim) | 0.73487 | 0.41645 | 1.765 | 0.07763 |
| education | 0.36002 | 0.08726 | 4.126 | <0.001 |
| Economic instability | -0.47642 | 0.16372 | -2.91 | <0.05 |

**S9 b)** The Buddhist and Muslim on the donation decision of the free donation game comparing with atheists respectively. Generalized linear multilevel model (binomial) was used with survey location as random effect.

|  | Estimate | SE | Z value | Pr(>\|z\|) |
| --- | --- | --- | --- | --- |
| (Intercept) | -0.66378 | 0.39732 | -1.671 | 0.0948 |
| Muslim(ref:atheist) | 0.16803 | 0.30279 | 0.555 | 0.5789 |
| Buddhist(ref:atheist) | 0.54699 | 0.35 | 1.563 | 0.1181 |
| education | 0.37114 | 0.07049 | 5.265 | <0.001 |
| Economic instability | -0.40912 | 0.14095 | -2.903 | 0.0037 |

**Dice Allocation Game Tables**

Supplementary Table 10

**The 120 rolls and P value of Chi-square test for each die.**

| Point | 1 | 2 | 3 | 4 | 5 | 6 | P value |
| --- | --- | --- | --- | --- | --- | --- | --- |
| Dice1 | 18 | 15 | 18 | 18 | 25 | 27 | 0.352494 |
| Dice2 | 25 | 21 | 16 | 21 | 17 | 21 | 0.753754 |
| Dice3 | 15 | 23 | 23 | 19 | 18 | 23 | 0.723098 |
| Dice4 | 21 | 15 | 14 | 29 | 19 | 23 | 0.176609 |
| Dice5 | 14 | 24 | 17 | 24 | 22 | 20 | 0.54224 |
| Theoretical value | 20 | 20 | 20 | 20 | 20 | 20 |  |

Supplementary Table 11

**Model selection for dice game decisions (predicting mean claim across communities).**

Variables used in the Control model are sex ratio (proportion of female participants in each community), average level of education and average economic instability. CS refers to community size, R refers to three principal components of religiosity. All of above are at community level.

|  | ***Models*** | ***K*** | ***AICc*** | ***ΔAICc*** | ***AICcWt*** | ***LL*** |
| --- | --- | --- | --- | --- | --- | --- |
| **Mean dice points** | Control | 5 | 51.33 | 0 | 0.86 | -17.94 |
|  | Control+CS | 6 | 55.70 | 4.38 | 0.1 | -17.65 |
|  | Control+R | 8 | 57.50 | 6.18 | 0.04 | -11.75 |
|  | Control+R+CS | 9 | 66.70 | 15.37 | 0 | -11.50 |

Supplementary Table 12

**Parameter estimates in control and full model of determinants of mean claim across communities. A permutation approach to linear models was used here with small sample size.**

|  | Control model | | Full model | |
| --- | --- | --- | --- | --- |
|  | Estimate | Pr(Prob) | Estimate | Pr(Prob) |
| Sex ratio | -0.5864 | 0.686 | -1.2705 | 0.1324 |
| Economic instability | 0.5207 | 0.706 | -0.1207 | 0.9608 |
| Education | -0.2618 | 0.448 | 0.4252 | 0.318 |
| Community size  (ref:small) |  |  | 0.1761 | 0.4516 |
| Religious practice |  |  | 0.5801 | 0.095 |
| Divine punishment/reward |  |  | 0.2252 | 0.6667 |
| Other supernatural belief |  |  | -1.6936 | 0.0164 |

Supplementary Table 13

**Another three measures of dishonesty that can be derived from our task. A first measure of dishonesty is whether having a report of 6 (No claim), which is most likely honest. Participants’ incentive is to claim 5 for maximizing their income, but dice results distribution show they were more willing to report 4 not 5, this provides our second measure of dishonesty: whether having a report of 4 (Second highest claim). Our third measure is whether having high claims for reported numbers 3,4,5.**

**Linear regression analysis with permutation test of three measures of dishonesty** **respectively as a function of community size and religiosity.** Sociodemographic controls include sex ratio, education level and economic situation. Significant effects are in bold.

|  | Dependent variable | | | |
| --- | --- | --- | --- | --- |
|  | No claim  (Number 6) | Second Highest Claim  (Number 4) | | High Claim  (Numbers 3,4,5) |
| Sex ratio | 0.030 | -0.185 | -0.154 | |
| Economic instability | 0.007 | -0.098 | 0.032 | |
| Education | -0.013 | 0.044 | 0.086 | |
| Community size  (ref:small) | -0.010 | 0.012 | 0.042 | |
| Religious practice | -0.025 | 0.074 | 0.118 | |
| Divine punishment/reward | 0.040 | 0.051 | 0.051 | |
| Other supernatural belief | **0.132** | -0.100 | **-0.229** | |

Supplementary References

1. Gächter, S., & Schulz, J. F. (2016). Intrinsic honesty and the prevalence of rule violations across societies. Nature, 531(7595), 1–11. https://doi.org/10.1038/nature17160

2. Average wage in Gansu province: <http://www.gstj.gov.cn/HdApp/HdBas/HdClsContentDisp.asp?Id=11628>

3. Households basic situation of residents in Gansu province (2016): http://www.gstj.gov.cn/HdApp/HdBas/HdClsContentDisp.asp?Id=11627

4. http://www.qhtjj.gov.cn/tjData/ydsj/

5. Per capita disposable income: <http://data.stats.gov.cn/tablequery.htm?code=AD0E>

6. <http://www.pewforum.org/religious-landscape-study/>
